# Distinct Evolutionary Signatures of Human Parainfluenza Viruses 2 and 4 Reveal Host Antagonism Divergence and Phylogenetic Discordance

**DOI:** 10.1093/molbev/msaf217

**Published:** 2025-09-10

**Authors:** Stephanie Goya, Alexander L Greninger

**Affiliations:** Department of Laboratory Medicine and Pathology, University of Washington Medical Center, 850 Republican St. S130, Seattle, WA 98109, USA; Department of Laboratory Medicine and Pathology, University of Washington Medical Center, 850 Republican St. S130, Seattle, WA 98109, USA; Vaccine and Infectious Disease Division, Fred Hutchinson Cancer Research Center, PO Box 19024, Seattle, WA 98109, USA

**Keywords:** parainfluenza virus, phylogenetic analysis, paramyxovirus, host antagonism, genomic characterization, orthorubulavirus

## Abstract

Human parainfluenza virus 2 (HPIV-2) and human parainfluenza virus 4 (HPIV-4) are significant but underappreciated respiratory pathogens, particularly among high-risk populations including children, the elderly, and immunocompromised individuals. In this study, we sequenced 101 HPIV-2 and HPIV-4 genomes from respiratory samples collected in western Washington State and performed comprehensive evolutionary analyses using both new and publicly available sequences. Phylogenetic and phylodynamic analyses revealed that both HPIV-2 and HPIV-4 evolve at significantly faster rates compared to the mumps virus, a reference human orthorubulavirus. Notably, while HPIV-2 demonstrated the highest evolutionary rates in the surface glycoprotein HN, consistent with humoral immune-driven selection, the innate immune antagonist V/P gene evolved fastest in HPIV-4. We identified a hypervariable region within the HPIV-4V/P protein (residues 35 to 75), which structural modeling placed in a loop overlapping a known interferon antagonism domain in other paramyxovirus V proteins, though HPIV-4 is functionally incompetent in this activity. Expanded phylogenetic analysis across the *Paramyxoviridae* family uncovered a striking evolutionary discordance: while the HN glycoprotein and L polymerase of HPIV-4 and its 2 closest bat-derived viruses clustered within the *Orthorubulavirus* genus, their nucleoprotein (N), phosphoprotein (P), matrix (M), and fusion (F) proteins formed a distinct lineage outside the *Rubulavirinae* subfamily. Together, these findings highlight the distinct evolutionary trajectories of HPIV-2 and HPIV-4, raise hypotheses around complex *Paramyxoviridae* zoonotic events including recombination-like patterns, and demonstrate limitations of current L protein-based taxonomic classification schemes.

## Introduction

Human parainfluenza viruses (HPIVs) are common respiratory pathogens affecting children, the elderly, and immunocompromised individuals worldwide (Wang et al. 2021). First identified in 1959, HPIVs are classified into 4 serotypes (HPIV-1 to HPIV-4) and belong to the *Paramyxoviridae* family. Despite their similar name, these viruses are classified into 2 distinct genera: HPIV-1 and HPIV-3 are classified within the genus *Respirovirus*, while HPIV-2 and HPIV-4 fall within the *Orthorubulavirus* genus (Rima et al. 2019). Due to its higher annual prevalence, HPIV-3 has captured most of the attention paid to human parainfluenza viruses and is considered a prototype pathogen for vaccine and monoclonal development against the paramyxoviruses, alongside HPIV-1 (Cassetti et al. 2022). In contrast, HPIV-2 and HPIV-4 have historically circulated at lower frequencies leading to limited research on their evolutionary dynamics. This is particularly true for HPIV-4 which has been historically underdiagnosed due to its exclusion from early direct fluorescent antigen panels and for which a complete genome sequence was not reported until 2009 (Lau et al. 2005; Yea et al. 2009).

In the most recent update by the International Committee on Taxonomy of Viruses (ICTV 2023), the HPIV-2 viral species was renamed as *Orthorubulavirus laryngotracheitidis*, and HPIV-4 as *Orthorubulavirus hominis* (Simmonds et al. 2024). HPIV-4 is further divided into 2 antigenic subtypes, HPIV-4A and HPIV-4B, which were originally distinguished by hemagglutination inhibition and neutralizing tests (Canchola et al. 1964 ). The *Orthorubulavirus* genus in the *Rubulavirinae* subfamily contains 8 ICTV-recognized species, of which HPIV-2, HPIV-4, and mumps virus (MuV) comprise significant human pathogens, while the remaining species have been detected in simians, bats, swine, and several other mammals (Liu et al. 2015). Three new orthorubulaviruses detected in bats, but not yet confirmed by ICTV, suggest that the number of members of this genus is still growing (Drexler et al. 2012; Hause et al. 2021; Wang et al. 2024). Phylogenetic analysis of the viral polymerase L amino acid sequence has traditionally been used to classify genera within the *Paramyxoviridae* family (Rima et al. 2019).

HPIVs are enveloped viruses with a negative-sense, single-stranded RNA genome. The genomes of HPIV-2 and HPIV-4 are approximately 15 and 17 kb in length, respectively, and share a conserved structure consisting of 6 genes: N, V/P, M, F, HN, and L. Among these, the fusion protein (F) and the hemagglutinin–neuraminidase (HN) glycoproteins serve as the attachment proteins and primary viral antigens (Marcink et al. 2021). The V/P gene is particularly intriguing, as it undergoes RNA editing to produce 2 distinct proteins: the phosphoprotein (P) that connects the nucleocapsid protein (N) and the RNA polymerase (L) to execute genome transcription and replication and the V protein that plays a key role in evading the host innate immune response (Nishio et al. 2005a; Motz et al. 2013; Abdella et al. 2020). This RNA editing involves the insertion of 2 guanosine (+GG) nucleotides by transcriptional slippage, which causes a frameshift that extends the product by 169 amino acids (Douglas et al. 2021). In rubulaviruses, the default product of the V/P gene is the V protein (229 to 230 aa), while the edited transcript produces the longer P protein (399 aa) (Kondo et al. 1990).

Across most paramyxoviruses, including HPIV-2, the V protein plays a critical role in early innate immune evasion by targeting transcription factors STAT1 and/or STAT2 for proteasomal degradation via recruitment of a DDB1-CUL4 E3 ubiquitin ligase complex and by blocking MDA5, a cytoplasmic sensor of viral double-stranded RNA, thereby effectively suppressing type I interferon responses (Nishio et al. 2005a; Motz et al. 2013; Douglas et al. 2021). However, while the HPIV-4V protein retains the ability to bind DDB1, it is broadly incapable of inhibiting the interferon response in human cells (Nishio et al. 2005b).

Due to their limited prevalence, the HPIV-2 and HPIV-4 genome evolution has been remarkably understudied to date. Here, we present the plurality of publicly available HPIV-2 and HPIV-4 genomes as well as the largest comparative evolutionary analysis of HPIV-2 and HPIV-4 at the genomic level. We identify unique evolutionary patterns in HPIV-4, particularly in the V/P gene, which exhibits an unexpectedly high evolutionary rate. Furthermore, phylogenetic analysis of *Rubulavirinae* viral subfamily suggests that HPIV-4 is more evolutionarily distinct than previously recognized.

## Results

### Current State of Genomic Epidemiology of HPIV-2 and HPIV-4

**Fig. 1.**
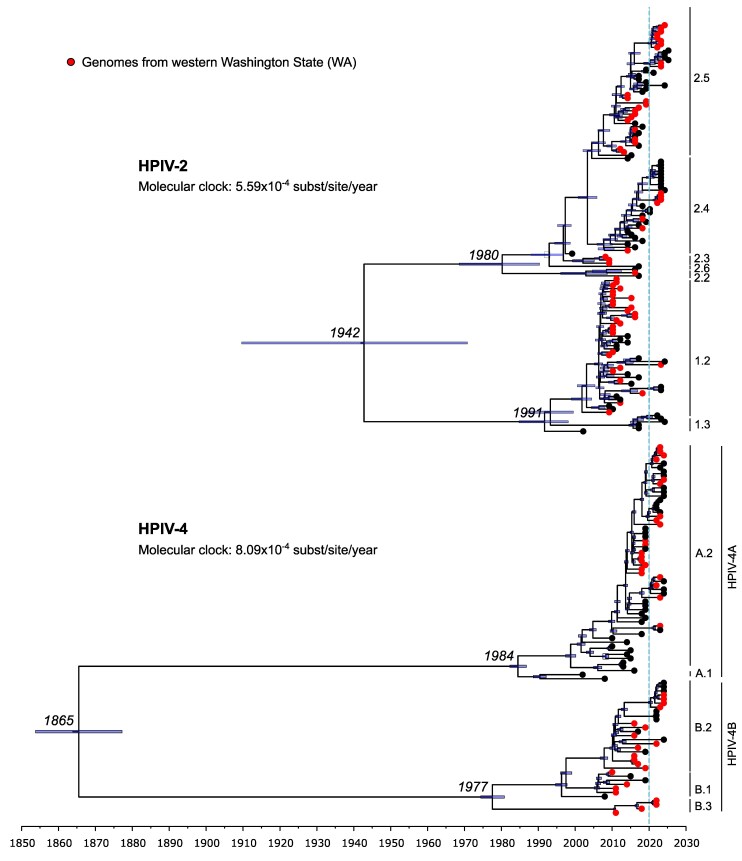
Bayesian phylodynamic trees based on HPIV-2 and HPIV-4 complete genomes. The estimated evolutionary rate in substitutions per site per year is indicated. Genomes from samples collected in western Washington State (WA) are marked with a red circle at the tree tips. A dotted light blue line separates the genomes collected after 2020. The scale bar represents time in years, and for relevant nodes, the time of the most recent common ancestor (tMRCA) is provided along with the tMRCA uncertainty (i.e. the 95% highest posterior density intervals) represented by blue bars. The HPIV-2 and HPIV-4 classification clade is indicated on the right side of the trees (Oh et al. 2021: 2015 to 2019).

Phylogenetic classification of HPIV-2 and HPIV-4 at the subspecies level is based on HN gene sequences and pairwise *p*-distance (Oh et al. 2021: 2015 to 2019). A total of 245 HPIV-2 and 349 HPIV-4 (193 HPIV-4A and 156 HPIV-4B) sequences were analyzed, with 50% of HPIV-2 and 27% of HPIV-4 HN sequences derived from complete genomes (supplementary fig. S1, Supplementary Material online). HPIV-2 phylogeny resolved into 2 major lineages including 3 (1.1 to 1.3) and 6 (2.1 to 2.6) clades, respectively, whereas HPIV-4A and HPIV-4B are classified into 2 (A.1 to A.2) and 3 (B.1 to B.3) clades (supplementary fig. S1, Supplementary Material online). Most clades persisted post-2020, except HPIV-2 2.2, 2.3, and 2.6 clades; HPIV-4A A.1; and HPIV-4B B.1. Clade 1.3 of HPIV-2 was not detected recently in Washington State but observed in Russia and China (Fig. 1). Phylogenetic congruence was observed in whole genome trees, though not all clades (HPIV-2 1.1 and 2.1) included complete genomes.

Since 2018, UW Virology contributed 42% HPIV-2 and 45% HPIV-4 genomes in INSDC databases as of April 16, 2025 (Fig. 1, supplementary figs. S2 and S3, Supplementary Material online). While airborne transmission supports the potential for global dispersal of HPIV-2 and HPIV-4, the available genomic data remain geographically skewed. Until more globally representative sampling is achieved, our understanding of global diversity will largely rely on the best available datasets. HPIV-2/4 genomes from Washington State were derived from 51 (HPIV-2) and 39 (HPIV-4) unique individuals that were 28% (HPIV-2) and 42% (HPIV-4) female, respectively, for those with demographic data available (supplementary table S1, Supplementary Material online). HPIV-2 sequences primarily came from younger individuals (median 8.5 versus 55.5 years, Wilcox test, *P* = 0.0003), likely due to differences in regional reference laboratory testing and culture isolation patterns over the past 15 years. No age difference was observed between HPIV-4A and HPIV-4B individuals (*P* > 0.05, supplementary table S1, Supplementary Material online). A notable case of a 16-year-old female with a prolonged HPIV-2 infection (82 days between first and last positive, Ct values 19.9 to 35.1) yielded 8 genomes clustering monophyletically within clade 2.5 and showing minimal intra-host evolution with a fixation of a single emergent synonymous mutation in the L gene over the 3 months (supplementary table S2, Supplementary Material online). Cross-sectional analysis also revealed intrasample variability in 1/10 HPIV-2, 3/6 HPIV-4A, and 1/5 HPIV-4B samples containing 1 to 2 intra-host single nucleotide variants (iSNVs, supplementary table S2, Supplementary Material online). While immune status data were unavailable, the observed patterns suggest limited intra-host diversity.

HN phylogenies revealed distinct evolutionary patterns between HPIV-4 and HPIV-2. The antigenically distinct HPIV-4 subtypes (4A and 4B) formed 2 well-separated clades with long branches, while HPIV-2 showed less divergence between its 2 main lineages (supplementary fig. S1, Supplementary Material online). As the primary target of humoral immunity (Suryadevara et al. 2024), HN glycoprotein typically exhibits high divergence; the smaller patristic distance between HPIV-2 clades suggests they remain genetically and antigenically similar, unlike HPIV-4 subtypes (supplementary fig. S1, Supplementary Material online). Bayesian phylodynamic analysis estimated HPIV-2 clades diverged ∼83 years ago (1942; 95% HPD: 1910-1971), while HPIV-4 subtypes separated ∼159 years ago (1865; 95% HPD: 1855-1879) (Fig. 1). At genomic level, HPIV-2 and HPIV-4 evolutionary rates fall within the expected range for single-stranded RNA viruses, but lower in HPIV-2 (5.59 × 10⁻⁴ substitution per site per year hereinafter ssy, 95% HPD: 4.72 × 10⁻⁴ to 6.59 × 10⁻⁴) than HPIV-4 (8.09 × 10⁻⁴ ssy, 95% HPD: 7.56 × 10⁻⁴ to 8.65 × 10⁻⁴).

### Evolutionary Rate Comparison Highlights Distinctive V/P Gene

**Fig. 2.**
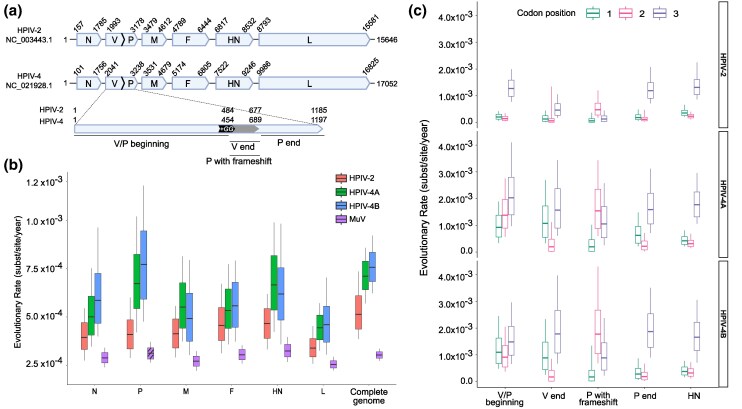
Comparison of the evolutionary rates of HPIV-2, HPIV-4A/B, and mumps virus. a) The genome organization of HPIV-2 and HPIV-4A reference strains is depicted along with the nucleotide number of the genome and gene borders. The multiple reading frames of the P/V gene are detailed at the bottom of the panel. The location of the transcriptional slippage is highlighted in black (+GG), and the location of the overlap between V and P sequences after the frameshift is highlighted in gray. b) The evolutionary rates of each gene and the complete genome from BEAST analysis are shown for HPIV-2, HPIV-4A, HPIV-4B, and the mumps virus. The box plot represents the 95% highest posterior density intervals where the horizontal bar represents the median value, and the vertical bars represent the complete range of values. The estimation of the evolutionary rate of the P gene in the mumps virus is indicated with a line pattern, as the tree likelihood parameter did not converge during the BEAST run. The y-axis minimum was set to 2 × 10^−4^ substitutions per site per year for visualization purposes. c) Evolutionary rates of the different sequence regions in the P/V gene indicated at the bottom of Fig. 2a for HPIV-2, HPIV-4A, and HPIV-4B subtypes.

HPIV-2 and HPIV-4 share the same genome organization, though HPIV-4 (17 kb) is longer than HPIV-2 (15 kb) mainly due to differences in the non-coding regions between the M-F, F-HN, and HN-L genes (Fig. 2a). The gene coding sequences vary by less than 25 nucleotide length between viral species, suggesting evolutionary constraint on the viral proteins.

Given HPIV-4 has a higher genomic evolutionary rate compared to HPIV-2, we examined evolutionary rates independently for each gene (Fig. 2b). HPIV-4A and HPIV-4B subtypes were analyzed separately to avoid confounding by antigenic divergence, and we included MuV genomic data from INSDC databases as a reference human orthorubulavirus. HPIV-2 exhibited relatively uniform evolutionary rates across genes. The HN gene showed the highest median rate (4.58 × 10^−4^ ssy), but this was statistically significant only when compared to the N gene and L gene, which had the slowest rate compared to any other gene (3.39 × 10^−4^ ssy) (Fig. 2b, supplementary fig. S4a, Supplementary Material online). MuV displayed lower evolutionary rates than both HPIV-2 and HPIV-4 (supplementary fig. S4b, Supplementary Material online), with HN (3.24 × 10^−4^ ssy) being the fastest-evolving gene and L the slowest (2.54 × 10^−4^ ssy) (Fig. 2b, supplementary fig. S4a, Supplementary Material online). Of note, the V/P gene of MuV analysis did not converge in the tree likelihood parameter during the analysis, so this result should be interpreted with caution.

In contrast, HPIV-4 showed its highest evolutionary rate in the V/P gene (HPIV-4A: 6.76 × 10^−4^ ssy, HPIV-4B: 8.65 × 10^−4^ ssy), followed by HN (HPIV-4A: 6.91 × 10^−4^ ssy, HPIV-4B: 7.12 × 10^−4^ ssy), with the L gene having the lowest rates (HPIV-4A: 4.74 × 10^−4^ ssy, HPIV-4B: 5.14 × 10^−4^ ssy) (Fig. 2b, supplementary fig. S4a, Supplementary Material online). Given the elevated evolutionary rate of V/P in HPIV-4, we further investigated its evolution using codon partitions across 3 regions: (i) the beginning of the gene where V and P sequences share the same coding frame, (ii) the segment from the transcriptional slippage (+GG insertion) to the end of the V gene, and (iii) the C-terminal region unique to the P gene (Fig. 2a). We also analyzed the second region based on the original sequence frame and considering the frameshift, using the HN gene as a control.

As expected, the HN gene showed a mutation profile with a highest rate at the third codon position, where synonymous mutations predominate (Fig. 2c). In HPIV-2, this elevated third-position rate pattern was observed in nearly all V/P gene regions, except in the frameshifted overlap, where the third position in the V coding frame corresponds to the second position for the P coding frame. In HPIV-4, the expected codon rate pattern was observed only in the functional C-terminus of the phosphoprotein. All other HPIV-4 V/P regions, especially in the frameshifted overlap, displayed high nonsynonymous rates. The initial V/P overlap region also showed elevated evolutionary rates at the first and second codon positions. Together, these findings suggest a high fixation rate of both silent and nonsynonymous mutations in the V protein, while the C-terminal region of the P protein remains conserved.

### V Protein Variability in HPIV-4

To explore how the higher evolutionary rates affect coding diversity of the HPIV-4 V protein, we evaluated alignments for specific variability hotspots. Residues spanning positions 35 to 75 of the V/P protein showed the greatest variability, both in the amino acid residues themselves and in their physicochemical properties, across both subtypes and within subtypes (supplementary fig. S5, Supplementary Material online). The overlapping V/P region, particularly the P protein frameshift segment, was even more variable in HPIV-4 than in HPIV-2 or MuV. Given the limited number of HPIV-4 sequences analyzed (*n* = 74 nonidentical sequences), additional sampling will likely reveal greater diversity. In contrast, the C-terminal region of the P protein was relatively conserved across all viruses. Selection pressure analyses revealed predominantly purifying selection across the V and P proteins of HPIV-2 and MuV, except for the V/P overlap region in the P reading frame, which exhibited a higher nonsynonymous mutation rate (dN), but without statistical support for positive selection (supplementary fig. S5, Supplementary Material online). In HPIV-4, elevated dN values were observed across the entire V/P protein, particularly within the hypervariable region; however, no statistically significant evidence of diversifying selection was detected. In contrast, an increase in sites under purifying selection was identified toward the C-terminal region of the HPIV-4 P protein.

**Fig. 3.**
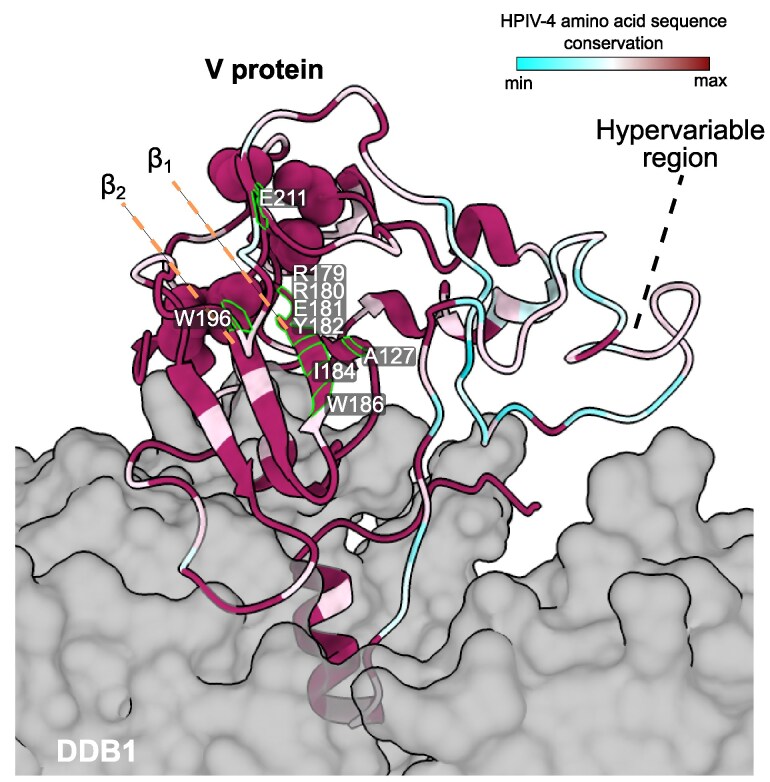
Structural modeling of the HPIV-4B V protein. The theorical 3-dimensional model was generated using SWISS-MODEL workspace for the sequence 086794/USA/2024 by homology with the crystal structure of the paramyxovirus V (PIV5, previously known as Simian Virus 5) protein in complex with DDB1 (PDB ID: 2B5L), since HPIV-4B V protein has previously been shown to retain DDB1 binding (Nishio et al. 2005b; Li et al. 2006). The model was visualized with ChimeraX-1.9. The structure is colored based on the amino acid sequence conservation of the HPIV-4 dataset using the AL2CO entropy-based calculation method. The V protein Cys-rich domain is highlighted as spheres. Residues A127, Y182, W186, W196, and E211 align with the conserved motif described in HPIV-2 (Nishio et al. 2005a), while residues R179, R180, E181, and I184 correspond to positions inferred with the PIV5V protein to interact with MDA5 (Motz et al. 2013).

To assess sequence conservation in the context of V protein structure, we generated a homology model based on the V protein of parainfluenza virus 5 (PIV5, formerly Simian Virus 5) in complex with DDB1, the closest *Orthorubulavirus* structure available in PDB. The hypervariable region (residues 35 to 75) corresponds to a loop connecting an alpha helix—implicated in DDB1 interaction—to the remainder of the protein structure (Fig. 3). At the C-terminus, residues essential for DDB1 interaction, along with the Cys-rich domain, were highly conserved across all HPIV-4 sequences. Interestingly, HPIV-4 also featured an additional cysteine at position 200 (within the Cys-rich domain). Conserved motifs, including the tryptophan motif Y182-(X)_3_-W186-(X)_9_-W196 within the B_1_ and B_2_ sheets, critical for V/STAT2 complex formation in HPIV-2, were also preserved. However, 2 phenylalanine residues experimentally shown in HPIV-2 to be essential for anti-interferon activity (F143 and F207) (Nishio et al. 2005a) have diverged in HPIV-4, corresponding to alanine (A127) and glutamic acid (E211), both well-conserved within HPIV-4. Critical B_1_/B_2_ sheet residues described in PIV5 to interact with MDA5 and inhibit ATPase hydrolysis and filament formation, thereby disrupting antiviral signaling (Motz et al. 2013), were also conserved in HPIV-4. Overall, within the species, the HPIV-4A/B V protein is well-conserved except for the hypervariable loop. The genetic and amino acid plasticity in this loop may reflect selective pressure for host adaptation and enhanced binding affinity, or alternatively, functional redundancy/inactivity that permits mutation accumulation without impacting the overlapping P protein or viral fitness.

### HPIV-4 Evolutionary Relationship Within Rubulavirinae Subfamily

**Fig. 4.**
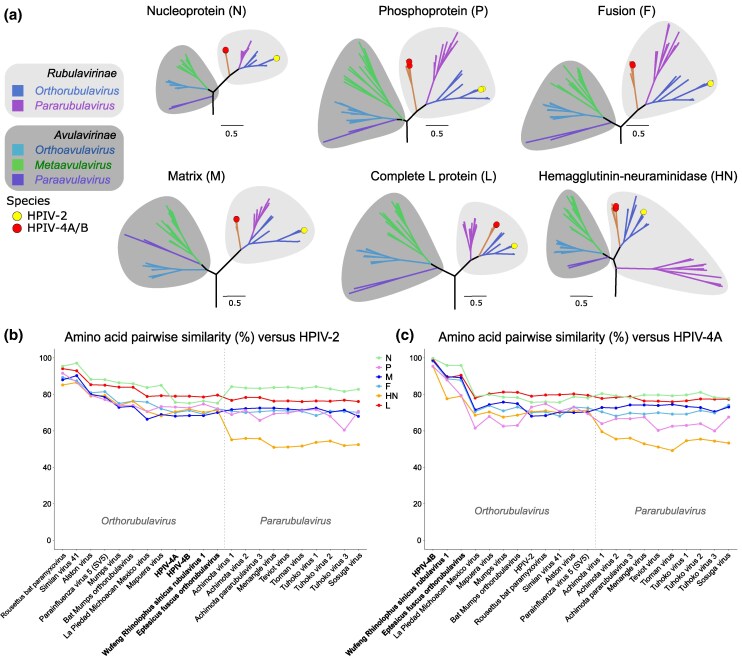
Phylogenetic trees of the *Rubulavirinae* and *Avulavirinae* subfamilies based on viral proteins. a) Maximum likelihood trees are shown for annotated protein sequences [N, P, M, F, receptor-binding protein (HN), and L] from representative *Paramyxoviridae* viral species from INSDC databases. The full version of each tree is available in supplementary figs. S6 and S7, Supplementary Material online as well as on GitHub (see Materials and Methods section). Tree branches are color-coded by genus, with genera belonging to any of the 2 taxonomic subfamilies highlighted in varying shades of gray. All trees are scaled using the same unit (0.5 substitutions per site). HPIV-4A/B are marked with red circles, and HPIV-2 with a yellow circle. b and c) Pairwise similarity plots based on the BLOSUM62 matrix between each viral protein of *Rubulavirinae* representative species against HPIV-2 (b) or HPIV-4A (c).

The distinct evolutionary pattern of the HPIV-4V/P gene prompted a more comprehensive phylogenetic analysis of individual genes across the *Paramyxoviridae* family. Phylogenies were generated for each structural protein using all available viral species (Fig. 4a; complete trees in supplementary figs. S6 and S7, Supplementary Material online). For the V protein, only viral species that encode it were included, as some paramyxoviruses instead express an alternative C accessory protein (supplementary fig. S8, Supplementary Material online).

Protein-specific phylogenies consistently showed that HPIV-4 is most closely related to 2 recently described bat viruses—Wufeng *Rhinolophus sinicus* rubulavirus 1 and *Eptesicus fuscus* orthorubulavirus—while HPIV-2 clustered with Simian parainfluenza virus 41 and *Rousettus* bat paramyxovirus, and MuV was most closely related to Bat mumps orthorubulavirus. These relationships suggest that human orthorubulaviruses have independent zoonotic origins, with only a limited range of mammalian hosts sampled to date.

Paramyxovirus species consistently clustered by taxonomic genus, though HPIV-4 displayed an unusual pattern (Fig. 4a). Due to its highly constrained structure/function, strong phylogenetic signal, and low evolutionary rate, the L protein has historically served as the basis for taxonomic classification within the *Paramyxoviridae*. Our L protein phylogeny confirmed the ICTV classification, showing HPIV-4 as closely related to other orthorubulaviruses but diverging first, as previously reported (Fig. 4a) (Rima et al. 2020). The HN phylogeny mirrored this topology (supplementary fig. S7, Supplementary Material online). The V protein tree showed a similar topology but with poor statistical support due to low phylogenetic signal, limiting definitive conclusions (supplementary fig. S8, Supplementary Material online). Interestingly, the phylogenies of the N, P, M, and F proteins indicated that HPIV-4 diverged earlier than the common ancestor of the *Orthorubulavirus* and *Pararubulavirus* genera (Fig. 4a, supplementary figs. S6 and S7, Supplementary Material online). Typically, such discrepancies in the HPIV-4 clade might suggest recombination with a breakpoint between the F and HN proteins; however, recombination is generally considered extremely rare or nonexistent in the *Paramyxoviridae* family (Larsen et al. 2022). Analysis of complete paramyxovirus genomes revealed no recombination hotspots, and the approximately unbiased (AU) test indicated that no alternative tree topology was significantly associated with the protein alignments, supporting the presence of genuine phylogenetic incongruence (supplementary table S3, Supplementary Material online). These findings suggest that different evolutionary pressures, such as viral host-jumping and adaptation, may be driving the varied phylogenetic topologies observed.

Additionally, in the HN phylogeny, the *Pararubulavirus* genus clustered earlier than expected relative to the *Avulavirinae* subfamily. The M protein tree placed the *Pararubulavirus* genus as a nested clade within *Orthorubulavirus*, although HPIV-4 branched earlier than both. Notably, Tupaia paramyxovirus (GenBank accession number AF079780.2), within the *Orthoparamyxovirinae* subfamily, was the only other viral species with inconsistent topology, clustering outside the *Narmovirus* genus in F and HN protein trees (supplementary figs. S6 and S7, Supplementary Material online).

Amino acid pairwise similarity analyses between HPIV-2, HPIV-4, and other representative *Rubulavirinae* species, based on the BLOSUM-62 matrix, showed a progressive decrease in similarity with more distantly related species with values approaching 70% (Fig. 4b). The L protein and nucleoprotein showed the highest similarity overall. Interestingly, compared to HPIV-2, HPIV-4 and its closely related bat viruses showed a notable decrease in similarity in the nucleoprotein, even when compared to pararubulaviruses. Overall, HPIV-4 exhibited lower pairwise similarity (below 80% amino acid identity) compared to both *Orthorubulavirus* and *Pararubulavirus* (Fig. 4c), except for the 2 closely related bat viruses, which shared 80% or higher similarity. The HN protein showed a substantial drop in similarity for both HPIV-2 and HPIV-4 when compared to pararubulaviruses (around 50%), consistent with their divergent clustering in the HN phylogeny.

Phylogenetic distance analysis among genera indicated that *Orthorubulavirus* and *Pararubulavirus* clades are more closely related to each other than to the HPIV-4 clade, consistent with the topology observed in the N, P, M, and F protein trees. However, in the L protein tree, the HPIV-4 clade exhibited greater divergence, with patristic distances between clades showing 0.59 amino acid substitutions per site compared to the *Orthorubulavirus* clade, 0.9 substitutions per site compared to the *Pararubulavirus* clade, but 0.49 substitutions per site between the *Orthorubulavirus* and *Pararubulavirus* genera (Table 1). These findings highlight the complexity of viral evolution and suggest that HPIV-2 and HPIV-4 may have evolved under distinct selective pressure, potentially involving multiple host species. These results underscore the importance of analyzing multiple viral proteins to fully understand the origins and evolutionary history of viruses.

**Table 1:** Patristic distances between nodes of the *Rubulavirinae* genera, indicated as *Orthorubulavirus*, *Pararubulavirus*, and the HPIV-4 clade from maximum likelihood trees

Protein sequence	*Orthorubulavirus* vs. *Pararubulavirus*	HPIV-4 clade vs. *Orthorubulavirus*	HPIV-4 clade vs. *Pararubulavirus*
N	**0**.**31**	0.82	0.87
P	**0**.**74**	1.14	1.46
M	Nested clade topology	0.60	
F	**0**.**71**	0.87	1.25
HN	1.47	**0**.**54**	1.74
L	**0**.**49**	0.59	0.90

In bold, the lower patristic distance of the phylogenetic tree constructed with each viral protein. Values were calculated as the median of 3 phylogenetic tree replicates and are indicated as substitutions per site.

## Discussion

Despite similarities in nomenclature, taxonomic classification, and virion structure, HPIV-2 and HPIV-4 are highly distinct viruses. In this study, we present new genomic data for HPIV-2 and HPIV-4 and provide a comprehensive evolutionary comparison. Our findings suggest that HPIV-4 has a distinct ancestral origin, differentiating it not only from HPIV-2 but also from the entire *Orthorubulavirus* genus. While phylogenetic analysis based on the L protein places HPIV-4 close to other orthorubulaviruses, its evolutionary trajectory differs when examining other viral proteins. Notably, although the HPIV-4 V protein retains a conserved domain that, in other viruses, has been associated with evasion of the interferon-induced antiviral response, the evolutionary rate of the V/P gene is unusually high, comparable to or exceeding that of receptor-binding proteins HN and F. These findings offer evolutionary correlates of unique epidemiological features of HPIV-4, such as lower hospitalization rate and less severe disease, as well as its biological traits like slower growth in cell culture (Canchola et al. 1964 ; Chellapuri et al. 2022).

Global data on the burden and prevalence of HPIV-2 and HPIV-4, in both asymptomatic and symptomatic individuals, remain limited (Wang et al. 2021). Recent studies from the United Kingdom indicate that HPIV-4 is detected more frequently than HPIV-2, with positive cases commonly associated with underlying medical conditions. While both viruses exhibit comparable severity, HPIV-4 is associated with a lower hospitalization rate (Maykowski et al. 2018; Chellapuri et al. 2022). HPIV-2 typically peaks in the fall, whereas HPIV-4 circulates year-round (Fry et al. 2006).

Historically, the HN gene has been the most widely used genomic region for studying parainfluenza virus diversity and for phylogenetic classification (Oh et al. 2021; Zhu et al. 2024). This focus has shaped much of the available genetic data, especially for HPIV-4, where partial or complete HN sequences predominate. While the HN gene offers a cost-effective marker that closely mirrors full-genome phylogenetic topology, relying solely on this region may obscure important aspects of viral evolutionary dynamics. As of 2025 April 16, only 50% of HPIV-2 and 27% of HPIV-4 HN sequences available in the INSDC databases correspond to complete genomes. Notably, 42% of HPIV-2 and 45% of HPIV-4 genomes were contributed by the University of Washington Virology laboratory. In the absence of a more globally representative surveillance effort, these datasets currently provide the best available window into HPIV-2 and HPIV-4 evolution. Future expansions in global sequencing efforts may refine or revise these findings.

Based on analysis of the complete genomes, we found that both HPIV-2 and HPIV-4 evolve at significantly faster rates compared to MuV, a reference human orthorubulavirus. HPIV-4 stands out with the highest evolutionary rate, a trend that persists even when its 2 antigenic subtypes (HPIV-4A and HPIV-4B) are analyzed separately. Interestingly, we found that the HPIV-4V/P gene exhibits an unusually high evolutionary rate comparable to that of the HN gene. This pattern is only seen in HPIV-4, as HPIV-2 and MuV have elevated evolutionary rates confined to their surface glycoproteins, which are under immune pressure. The elevated evolutionary rate in HPIV-4 likely corresponds to nonsynonymous changes located in a hypervariable region (residues 35 to 75) within the overlapping segment of the V and P proteins. Structural homology modeling of the HPIV-4V protein shows that this region lies within a loop connecting the conserved DDB1-interacting alpha helix to the rest of the protein (Li et al. 2006). This interaction is functionally important, as the paramyxovirus V protein hijacks the DDB1-Cul4A ubiquitin ligase complex to target STAT proteins for degradation, thereby antagonizing the interferon response (Li et al. 2006). In HPIV-4, critical residues within the alpha helix, cysteine-rich domain, and tryptophan-rich motif—critical for blocking interferon responses—are conserved. However, 2 residues previously identified in HPIV-2 (Phe207 and Phe143) (Nishio et al. 2005a) have been inferred to change into Glu211 and Ala127 in HPIV-4.

Nonetheless, the HPIV-4 V protein appears functionally deficient. Although it binds DDB1, Cul4A, STAT1, and STAT2, it does not reduce STAT1/2 levels or effectively suppress host interferon responses (Nishio et al. 2005b). This deficiency has been attributed to 3 regions within the HPIV-4 V protein (residues 32 to 45, 143 to 164, and 200 to 212), which overlap with the hypervariable loop we identified. Interestingly, mutations in the V protein of parainfluenza virus 5 have been shown to restrict its host range from humans to mice (Parisien et al. 2002). Based on this, we hypothesize that HPIV-4 may still be adapting to human hosts and that, over time, its V protein could evolve increased affinity and functionality in humans.

Phylogenetic analysis of the *Paramyxoviridae* family further supports this hypothesis. HPIV-4 clusters closely with 2 bat-derived viruses, while MuV shares ancestry with another bat virus, and HPIV-2 is most closely related to simian and bat viruses (Nishio et al. 1990; Hause et al. 2021; Paskey et al. 2023). These bat-borne orthorubulaviruses have all been isolated from phylogenetically disparate bat species, suggesting a broad range of evolutionary pressures for innate immune antagonism. Thus, comparison among the human orthorubulaviruses should be approached cautiously, as they do not share the same evolutionary pathways and have undergone different selective pressures. Alternatively, the V protein may serve a function yet to be determined or may not be essential for the HPIV-4 life cycle, possibly representing a vestige of host-jumping. If so, an uncharacterized viral strategy might have rendered the V protein redundant, allowing accumulation of mutations in regions that do not affect the function of the overlapping P protein without fitness cost. In any case, the inability of the V protein to evade interferon likely contributes to HPIV-4's poor growth in cell culture (Lau et al. 2005). Moreover, in other orthorubulaviruses such as HPIV-2, MuV, and parainfluenza virus 5, the V protein also inhibits genome replication by interacting with the L protein via its C-terminal domain (Nishio et al. 2008).

Unusually, the phylogeny of HPIV-4 and its 2 related bat-borne viruses diverges sharply from the canonical paramyxovirus pattern. While most viral species cluster within their defined genus based on the L protein, HPIV-4 clade clusters outside the *Rubulavirinae* subfamily in trees constructed from N, P, M, and F protein trees. In other viral families, such a phylogenetic pattern might indicate recombination with a breakpoint between F and HN genes. However, recombination is thought to be essentially impossible in paramyxoviruses, supporting the hypothesis that HPIV-4 evolutionary history was shaped by strong selective pressures associated with host-jumping events (Han and Worobey 2011; Larsen et al. 2022).

Finally, while the full-length L protein sequence is the current gold standard for taxonomic classification, our study highlights the importance of incorporating multiple viral proteins into evolutionary analyses. Relying solely on a single gene may obscure the complexity of viral adaptation and zoonosis. Comprehensive analyses that include as many viral proteins as possible may help clarify patterns of host tropism, immune pressure, and sequence–structure constraints. In this context, Vanmechelen et al. (2023) proposed a major restructuring of the *Paramyxoviridae* family based on phylogenetic analysis of a concatenated alignment of the 6 major paramyxoviral proteins (N, P, M, F, receptor-binding protein such as HN, L). Using pairwise patristic distance thresholds, they defined 5 new subfamilies, 6 new genera, and 71 new species. While the new framework provides much-needed standardization for defining *Paramyxoviridae* taxonomy, concatenating viral proteins can mask the complex evolutionary history of HPIV-4 and its bat-derived virus ancestors. Phylogenetic incongruence across multiple protein trees remains the best way for identifying recombination-like events (Esquivel Gomez et al. 2024), though we believe further paramyxovirus mammal surveillance is needed to contextualize our findings. Unfortunately, the absence of intermediate host ancestors complicates evolutionary reconstruction, as viral discovery has been biased toward human, bat, and avian hosts. Expanding respiratory virus surveillance in other mammals is critical for identifying the closest relatives of human-infecting viruses. As previously discussed by Larsen et al. (2022), it is also essential to determine whether these mammal-borne viruses cause disease in their natural hosts. In their study in Arizona, USA, Larsen and colleagues found no overt signs of disease in bats and rodents carrying novel *Orthoparamyxovirinae* viruses. Understanding the pathogenic potential in reservoir species may illuminate the steps involved in the transition to human infectivity.

## Materials and Methods

### Sample Collection

In this study, we analyzed genomic data from human parainfluenza virus (HPIV) isolates obtained from 2 sources: specimens from the University of Washington (UW) Virology Division respiratory virus biobank (collected 2009 to 2019) and recent clinical samples (collected 2022 to 2024).

Biobank isolates consisted of deidentified respiratory specimens that tested positive for HPIV-2 or HPIV-4 by reverse transcription quantitative real-time PCR (RT-qPCR), as previously described (Weinberg et al. 2013). Recent clinical samples include a random surveillance in 2 periods. From November to December 2022, we screened 8,356 respiratory samples collected at UW Medicine COVID-19 community testing sites, detecting 27 HPIV-2 and 19 HPIV-4 cases, of which 15 HPIV-2 and 12 HPIV-4 samples with cycle threshold (Ct) values <33 were selected for genome sequencing. Between December 2022 and November 2024, we collected remnant respiratory samples tested by the UW Virology Laboratory using the FDA-authorized Panther Fusion Paraflu Assay (Hologic). Of 253 samples analyzed, 34 HPIV-2 and 27 HPIV-4 were detected, of which 23 HPIV-2 and 17 HPIV-4 samples had a Ct value of <33.

The temporal distribution of positive specimens includes 2 to 23 genomes per year from 2009 to 2024, except for 2020 and 2021, during which no specimens were available (supplementary table S2, Supplementary Material online). This study was approved by the UW Medicine Institutional Review Board, with a consent waiver (STUDY00000408).

### HPIV-2 and HPIV-4 Genome Sequencing

Virus RNA was extracted using the Quick-RNA Viral Kit (Zymo Research). Genome sequencing was performed using 2 methods: samples were sequenced by metagenomic RNA sequencing, as previously described (Greninger et al. 2017), while 13 samples were sequenced using hybridization capture sequencing with the QIAseq xHYB Respiratory Panel (Qiagen) (supplementary table S2, Supplementary Material online). Sequencing was performed as either 2 × 100 bp or 1 × 100 bp run on an Illumina NextSeq200 or NovaSeq1000 sequencer.

The resulting FastQ files were processed using the REVICA pipeline (https://github.com/greninger-lab/REVICA-STRM). Briefly, REVICA trims adapters and filters reads based on quality using FASTP v 0.23.4 and then assembles the genome by mapping the reads against a reference database of seasonal respiratory virus genomes to find the most suitable reference with BWA-MEM v2.2.1(Li 2013). Consensus genomes were generated using iVar v1.3.1 if the assembly covered above 60% of the reference sequence with a minimum depth of 5× (Castellano et al. 2021). Consensus genomes were compared with de novo assembly contigs generated with metaSPAdes v4.0.0 to confirm the absence of bioinformatic artifacts due to reference-based assembly (Nurk et al. 2017). Mapping assemblies were visualized using Integrative Genomics Viewer (IGV) (Robinson et al. 2011). The presence of intrasample nucleotide variants (iSNVs) was assessed with iVar v1.3.1 in positions with a minimum depth of coverage of 50× and detection of iSNV with frequencies above 10%. Location of the iSNVs used genome coordinates of the reference genomes NC_003443.1 for HPIV-2, NC_021928.1 for HPIV-4A, and KY629773.1 for HPIV-4B.

### Phylogenetic and Phylodynamic Analyses

#### Within Viral Species Analyses: HPIV-2, HPIV-4, and MuV

Complete genomes of HPIV-2, HPIV-4, and MuV obtained from human clinical samples were downloaded on 2025 February 25, from NCBI GenBank and combined with the newly sequenced HPIV genomes from Washington State (WA). MuV was included as an *Orthorubulavirus* control for comparison with HPIV findings. Nucleotide sequences were aligned using MAFFT v7.490 and visualized with Aliview v1.28 to detect and correct alignment errors (Katoh et al. 2002; Larsson 2014). Maximum likelihood trees were inferred with IQ-TREE v2.1 using the SH-aLRT test (1,000 replicates) and UFBoot2 method (1,000 replicates) to evaluate the reliability of phylogenetic clades (Hoang et al. 2018; Minh et al. 2020). Clades were considered statistically supported if UFBoot2 ≥ 90% and SH-aLRT ≥ 80%. Phylogenetic trees were visualized using FigTree v1.4.4 (https://github.com/rambaut/figtree).

Root-to-tip temporal stringency correlation of viral sequences was assessed with Clockor2 (Featherstone et al. 2024). Outliers that did not follow the molecular clock inferred with Clockor2 were identified and removed before further analysis. The evolutionary rate was estimated using BEAST2 v2.7.5 using a strict molecular clock for root-to-tip correlation coefficients above 0.8; otherwise, relaxed molecular clock models were used (Bouckaert et al. 2019). Specifically, HPIV-2 was analyzed under a relaxed lognormal clock, HPIV-4 and MuV used a strict clock for the complete genome dataset and an optimized relaxed clock for gene-specific datasets. In addition, the tree priors were selected based on the results of the nested sampling test, with HPIV-4A/B using the exponential population model, and HPIV-4A and HPIV-B independently as well as MuV were analyzed using a constant population model and HPIV-2 with a Bayesian coalescent skyline model. The convergence of the BEAST inference was evaluated by examining the effective sampling size (ESS) and the highest posterior density interval (95% HPD) after a 10% burn-in using Tracer v1.7. The maximum clade credibility tree (MCCT) was summarized using TreeAnnotator, and clades were considered supported if the posterior probability was ≥0.8. Selection pressure was assessed using the HyPhy software package v2.5.75, employing the fixed-effects likelihood (FEL) (Pond and Frost 2005) method with a significance threshold of *P* < 0.1, and the Fast-Unconstrained Bayesian AppRoximation (FUBAR) (Murrell et al. 2013) method with a posterior probability of >0.9. Results were visualized using the HyPhy Vision web interface (http://vision.hyphy.org).

#### Within Taxonomic Family Analyses: HPIV-4 in the Context of Rubulaviruses

Complete genomes of representatives of each viral species within the *Paramyxoviridae* family shared at the International Committee on Taxonomy of Viruses (ICTV) resources (accessed on 2025 February 25) were downloaded from NCBI GenBank (Rima et al. 2020). NCBI blastp search based on the N and the L protein of HPIV-4, HPIV-2, and MuV was performed to identify recently discovered paramyxoviruses not reported in ICTV with an identity above 40% and were included in the analysis. Amino acid sequences of viral proteins of each representative viral species were downloaded from NCBI GenBank. Sequences were aligned using MAFFT-DASH, which integrates sequence and structural alignment (Rozewicki et al. 2019). Alignments were visually inspected with Aliview v1.28 and very low homology regions at the end of the alignments were trimmed resulting in alignments ranging from 231 to 2,283 amino acids in length (alignments available at https://github.com/greninger-lab/orthorubulavirus_HPIV/). Maximum likelihood trees were inferred from each amino acid alignment using IQ-TREE v2.1 with the UFBoot2 method (1,000 replicates). The approximately unbiased (AU) test was performed for each protein alignment to evaluate all inferred trees as alternative phylogenetic hypotheses using 10,000 bootstrap replicates (Shimodaira 2002). In addition, to confirm maximum likelihood topologies, we also inferred Bayesian trees using MrBayes v3.2.7 (Ronquist et al. 2012). Bayesian consensus trees were built from 2 parallel runs with 1 Monte Carlo Markov chain (MCMC) for 600,000 generations with trees sampled every 6,000 generations with a predetermined set of fixed amino acid rate matrices. MCMC convergence was assessed in TRACER v.1.5, and the initial 10% of the run was discarded as burn-in. Consensus trees were visualized with FigTree v.1.4.4, with clades having posterior probability <0.6 collapsed. For all paramyxoviruses, complete genome sequences were downloaded and aligned using MAFFT (L-INS-i algorithm). Recombination analysis was then performed with the Recombination Detection Program (RDP5) using the RDP, GENECONV, BootScan, MaxChi, Chimaera, SiScan, and 3Seq methods (Martin et al. 2021). A recombination hotspot was considered supported when detected by 4 or more methods.

### Mapping Amino Acid Conservation into the 3D Structure of Paramyxovirus V Protein

The V protein sequence from HPIV-4B isolate 086794 (GenBank accession number PV224552.1) collected from western Washington in 2024 was used as a query sequence for structural homology modeling in the online SWISS-MODEL server (Waterhouse et al. 2018). The structural alignment with a global model quality estimation (GMQE) score of 0.42 was obtained using PDB template 2B5L (method X-ray 2.8 Å) with 33.5% amino acid identity. 2B5L is the previously determined crystal structure of DDB1 in complex with parainfluenza virus 5 (previously known Simian Virus 5) V protein, a model *Orthorubulavirus* (Li et al. 2006). ChimeraX-1.9 was used to visualize the HPIV-4 amino acid sequence conservation using the AL2CO entropy-based calculation method (Pettersen et al. 2021).

## Supplementary Material

msaf217_Supplementary_Data

## Data Availability

Sequencing data and consensus genomes are available under BioProject PRJNA1027483, with accession numbers listed in supplementary table S2, Supplementary Material online. Phylogenetic trees and BEAST analysis are available online at https://github.com/greninger-lab/Orthorubulavirus_HPIV.
