# Supplementary material for: Distinct Evolutionary Signatures of Human Parainfluenza Viruses 2 and 4 Reveal Host Antagonism Divergence and Phylogenetic Discordance
Source: Mol Biol Evol. 2025 Sep 10;42(10):msaf217. doi: 10.1093/molbev/msaf217 (PMC12492262; doi:10.1093/molbev/msaf217)

Supplementary figure 1. Maximum likelihood tree of the HN gene from new sequences generated in this study and HN sequences available in NCBI GenBank (from partial or complete genomes). The HN sequences from complete genomes are indicated in bold. Colored tip circles indicate year of sample collection. The symbol * indicates longitudinally sampled genomes. NCBI accession numbers are indicated in the tip names, together with the country and year of sample collection. Monophyletic clades of HPIV-4 subtypes A and B are highlighted in light blue and green, respectively. Genotypes are indicated at the right of each clade. Phylogenetic trees were scaled to be the same scale unit (0.02 substitutions per site).

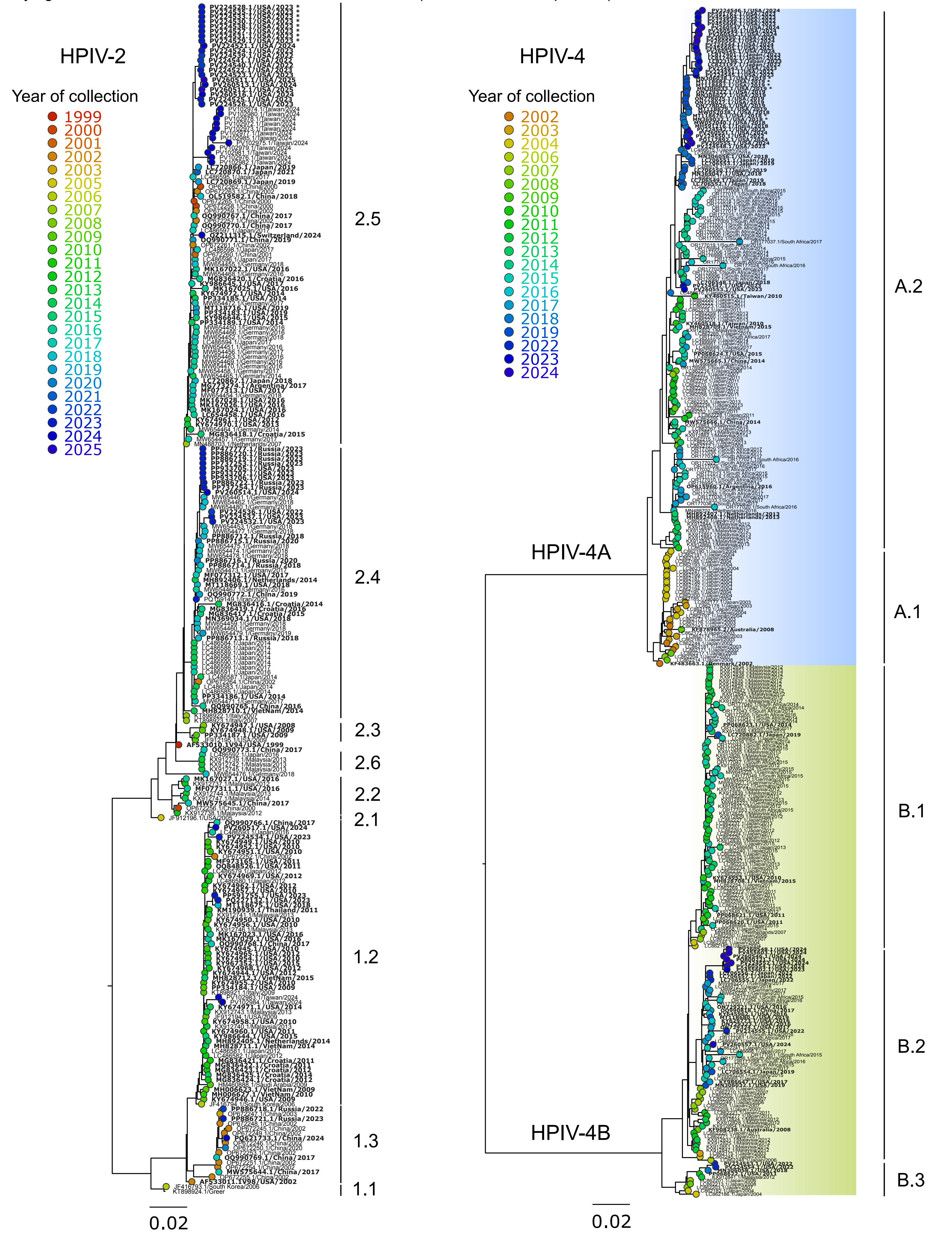

Supplementary figure 2. Bayesian phylodynamics tree based on HPIV-2 complete genomes. The estimated evolutionary rate in substitutions per site per year is indicated. Genomes from clinical samples collected after the year 2020 are indicated in red. Genomes from samples collected in western Washington State (WA) are marked with a black circle at the tree tips. The scale bar represents time in years, and for relevant nodes the time of the most recent common ancestor (tMRCA) is provided. The tMRCA uncertainty (i.e., the 95% highest posterior density intervals) is represented by blue bars. The HPIV-2 classification clade is indicated on the right side of the tree. The symbol * indicates longitudinally sampled genomes.

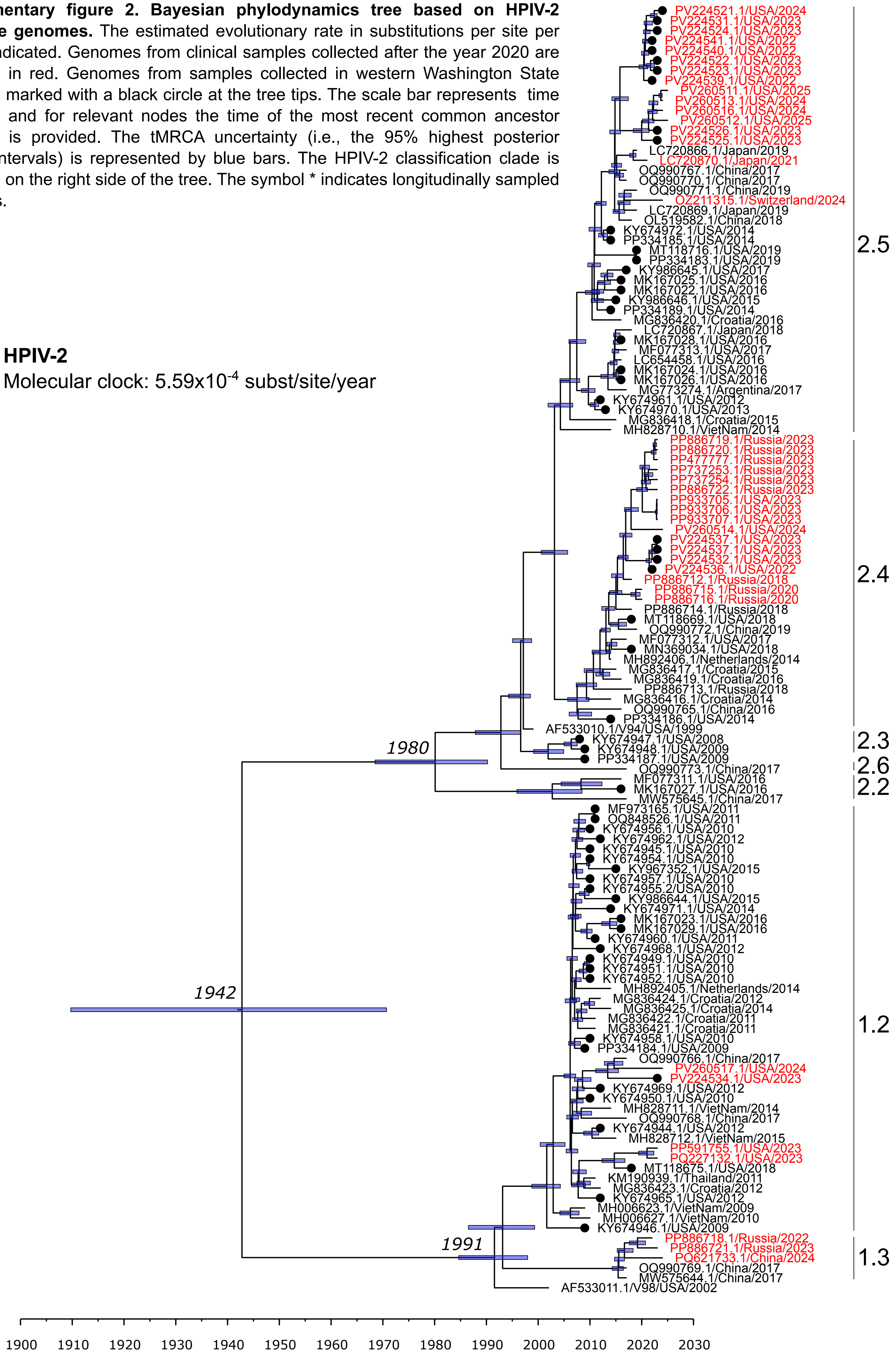

Supplementary figure 3. Bayesian phylodynamics tree based on HPIV-4 complete genomes. The estimated evolutionary rate in substitutions per site per year is indicated. Genomes from clinical samples collected after the year 2020 are indicated in red. Genomes from samples collected in western Washington State (WA) are marked with a black circle at the tree tips. The scale bar represents time in years, and for relevant nodes the time of the most recent common ancestor (tMRCA) is indicated. The tMRCA uncertainty (i.e., the 95% highest posterior density intervals) is represented by blue bars. The HPIV-4 classification clade is indicated on the right side of the tree.

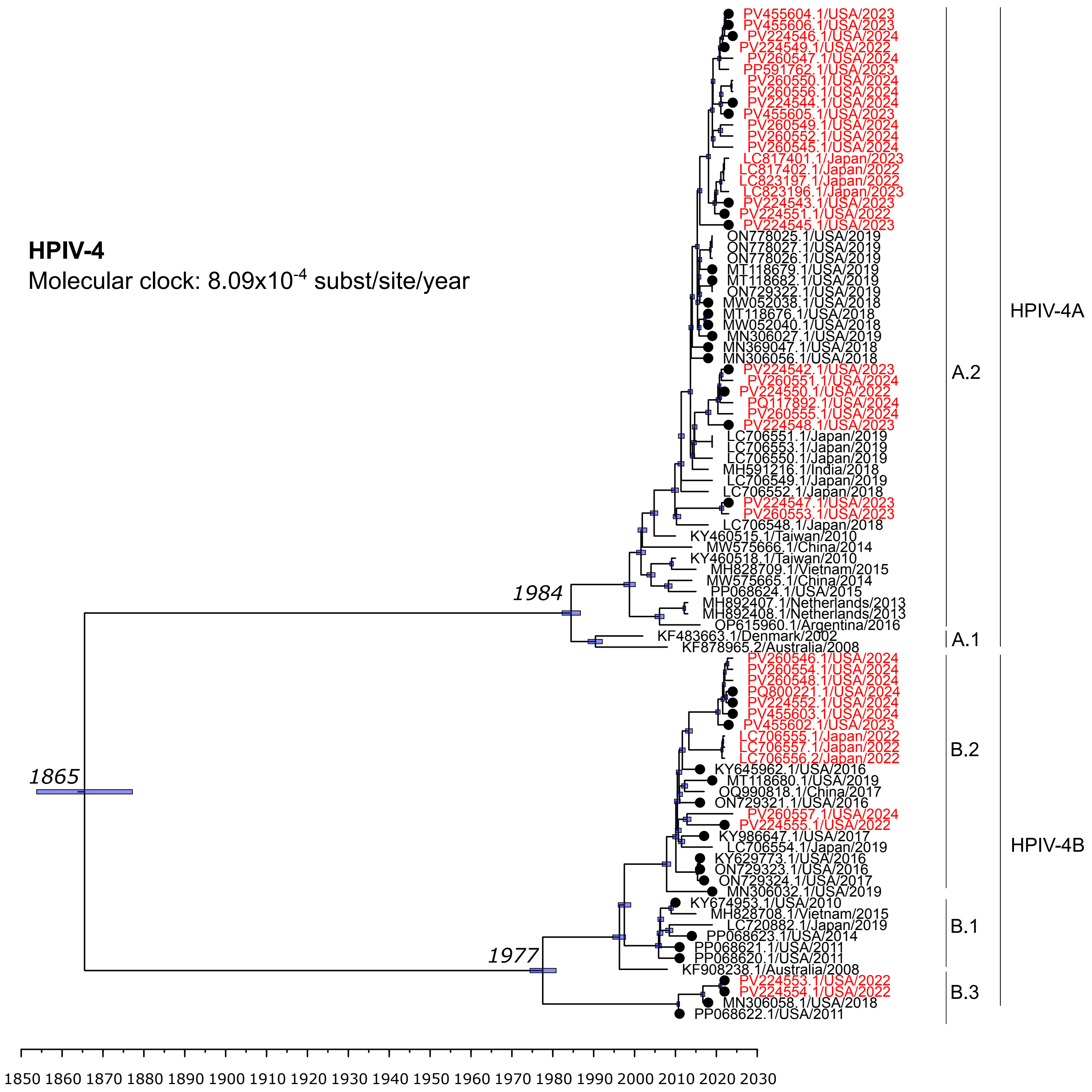

Supplementary figure 4. Evolutionary rate ratio of HPIV-2, HPIV-4 and mumps virus. A) Pairwise comparisons of gene-specific evolutionary rates within each virus. The color matrix shows the marginal posterior probability that the rate of a given gene (row) is higher than that of another gene (column). Probabilities were estimated using BEAST2 by comparing marginal posterior distributions across MCMC samples. A comparison is considered statistically supported if the 95% highest posterior density (HPD) of Pr(rate a > rate b | data) exceeds 0.95 (blue) or is below 0.5 (light blue). Comparisons not meeting these thresholds are considered inconclusive. Note that the matrix is not symmetric, as it reflects directional probability comparisons (row > column). B) Cross-species comparisons of homologous gene rates. For each gene, the posterior distribution of rate ratios (virus A / virus B) was estimated from BEAST2 MCMC samples. Blue dots represent the posterior median of the rate ratio, with blue lines indicating the 95% HPD interval. The red dashed line at 1 indicates equal rates; HPD intervals that do not include 1 reflect statistically supported differences.

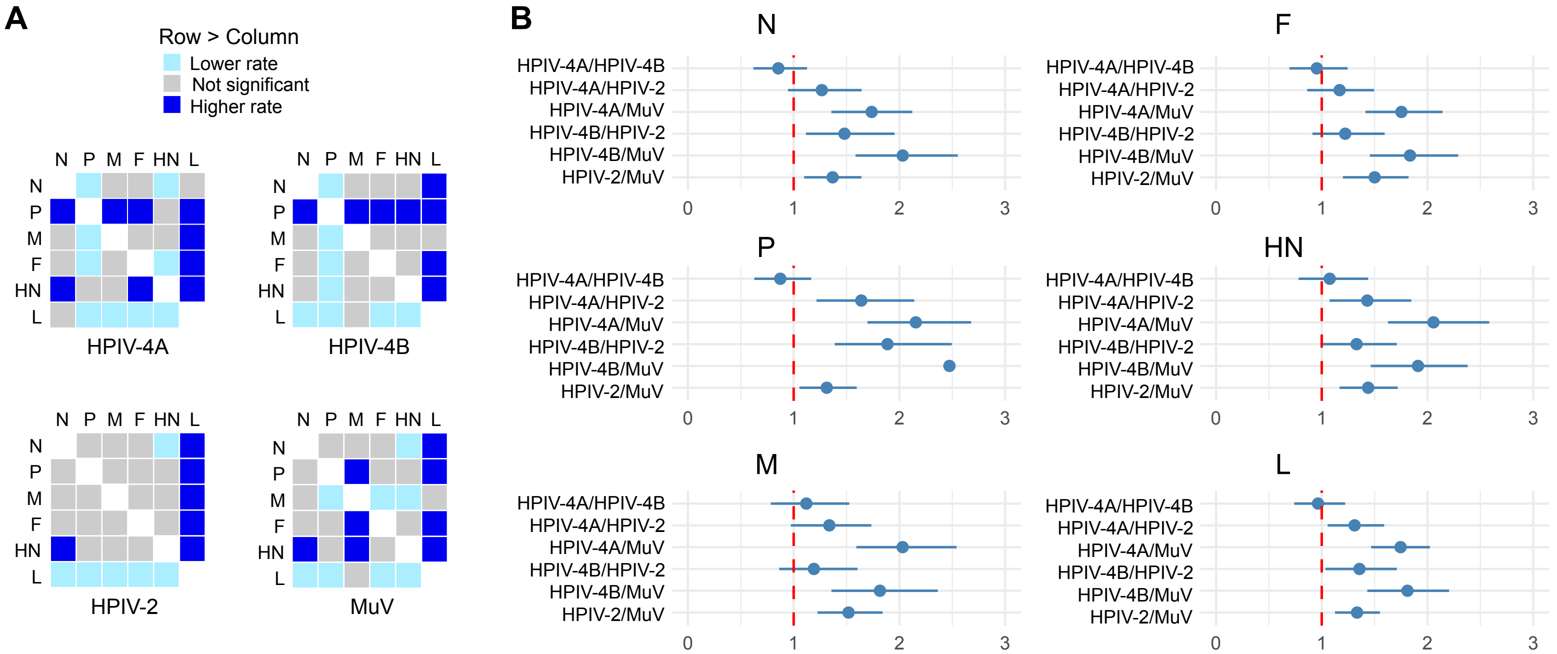

Supplementary figure 5. Conservation of the V and P protein viral datasets. Sequence logos show amino acid frequency per position for the V and P proteins of human parainfluenza virus 2 (HPIV-2), human parainfluenza virus 4 (HPIV-4), and mumps virus (MuV), using 98 HPIV-2, 74 HPIV-4, and 221 MuV non-identical sequences. Amino acids are colored by hydrophobicity (red = most hydrophobic; blue = most hydrophilic). Below each position, bar plots display non-synonymous (dN, green) and synonymous (dS, yellow) substitution rates. Sites under positive or purifying selection are marked with asterisks (*), based on FEL ($p < 0.1$) and FUBAR (posterior probability > 0.9); diversifying selection is marked above dN bars and purifying selection above dS bars. The overlapping region of the V and P genes resulting from the insertion of two guanosines is shaded in grey. Selection analyses in this region are shown specifically for the V protein.

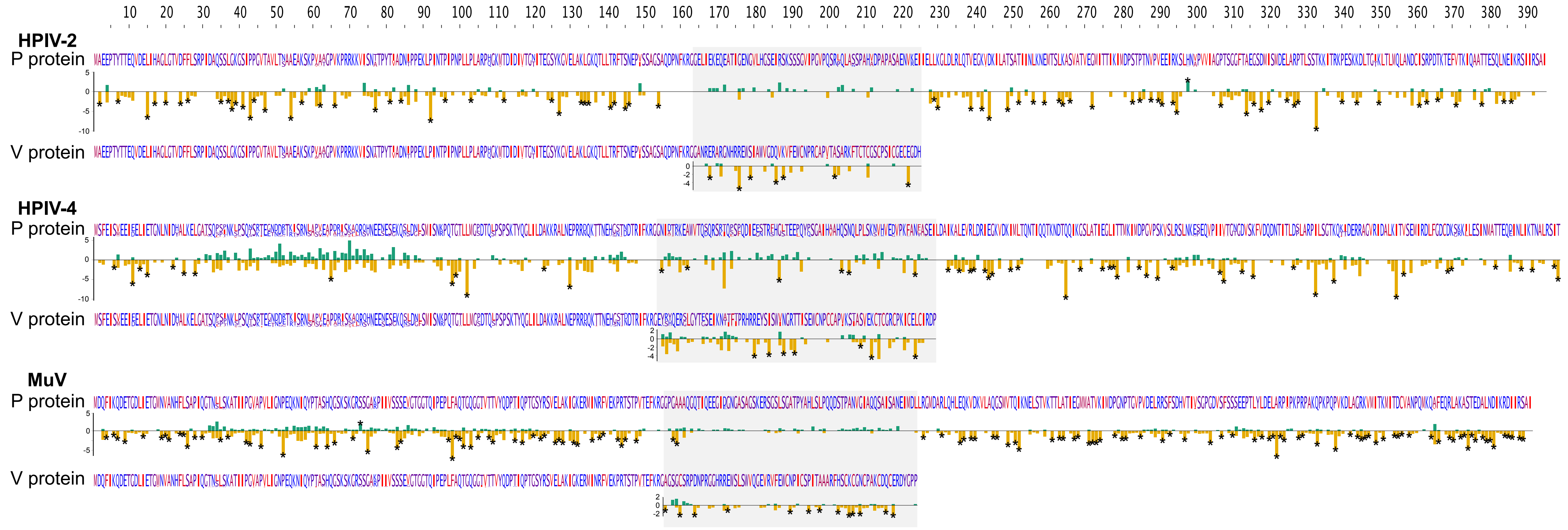

Supplementary figure 6. Maximum Likelihood Trees of the Paramyxoviridae Family Inferred with N, P, and M Amino Acid Sequences. Representative *Paramyxoviridae* species from NCBI GenBank were included, with each tree tip accompanied by its GenBank accession number. Annotated amino acid sequences were retrieved from NCBI records. Tree tips are color-coded by genus, and subfamilies are highlighted in colored background. Black dots on certain nodes defining subfamily or genera indicate statistical support above 80% SH-aLRT and 90% ultrafast bootstrap. The scale represents amino acid substitutions per site. Of note, Eptesicus fuscus orthorubulavirus (GenBank ID: MZ355765.1) was reported with a partial N protein, however, protein homology alignment suggests that only the first four amino acids are missing.

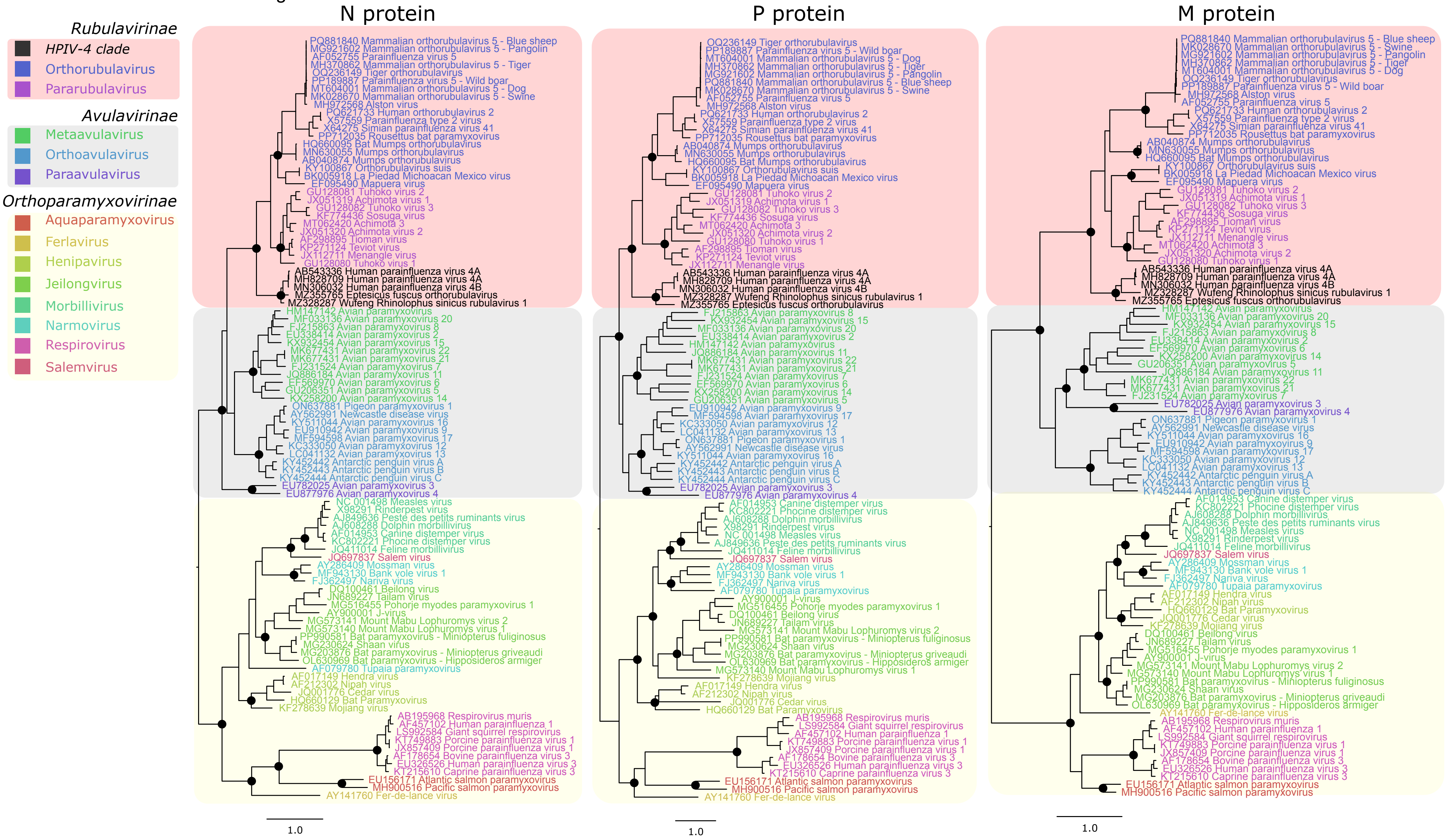

Supplementary figure 7. Maximum Likelihood Trees of the Paramyxoviridae Family Inferred with F, HN and L Amino Acid Sequences. Representative *Paramyxoviridae* species from NCBI GenBank were included, with each tree tip accompanied by its GenBank accession number. Annotated amino acid sequences were retrieved from NCBI records. Tree tips are color-coded by genus, and subfamilies are highlighted in colored background. Black dots on certain nodes defining subfamily or genera indicate statistical support above 80% SH-aLRT and 90% ultrafast bootstrap. The scale represents amino acid substitutions per site.

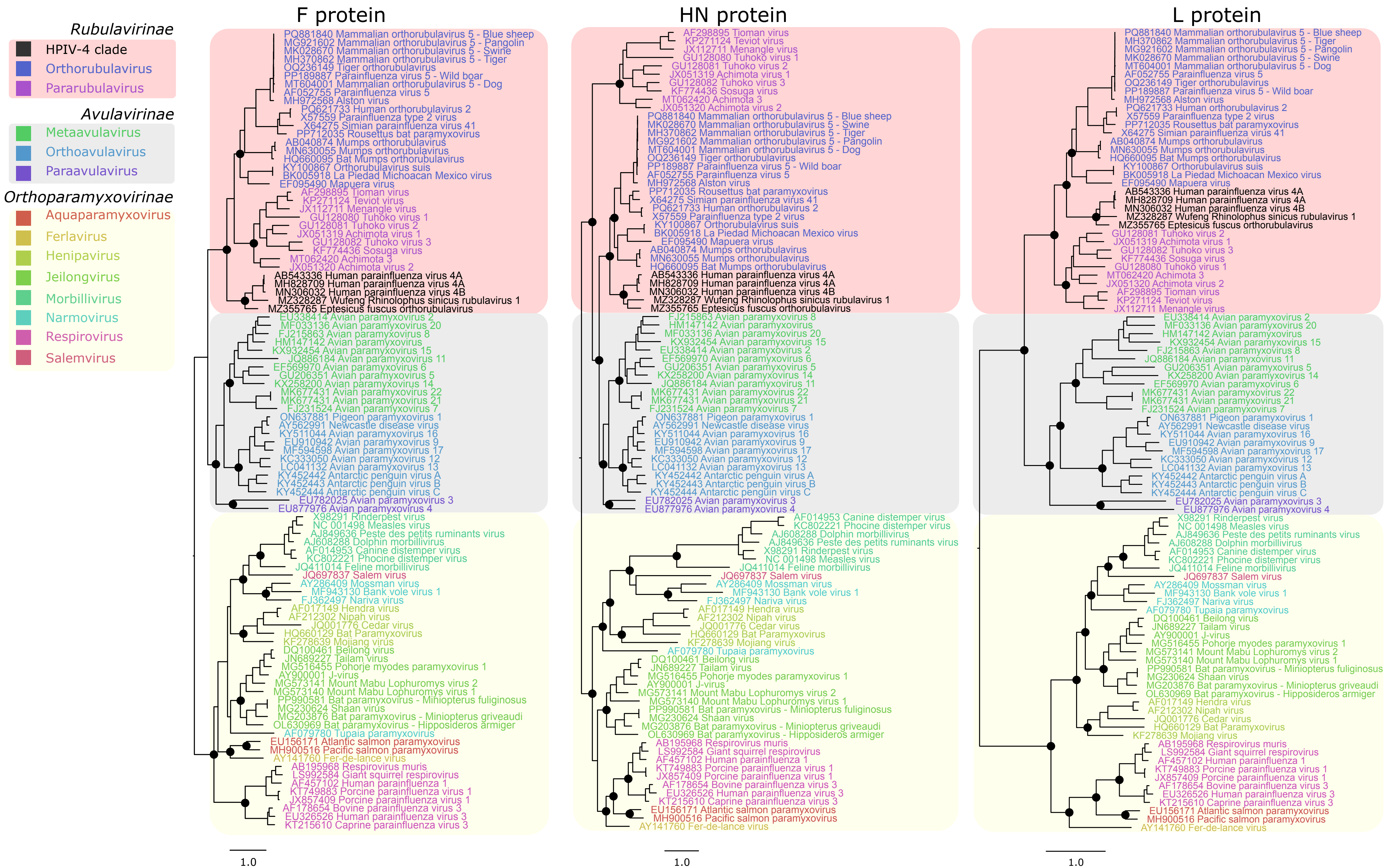

Supplementary figure 8. Maximum Likelihood Trees of Paramyxovirus V Protein Amino Acid Sequences. A) Sequences from representative virus species downloaded from NCBI GenBank were included, with each tree tip accompanied by its GenBank accession number. Tree tips are color-coded by genus, and subfamilies are indicated in the legend. Black dots on certain nodes defining subfamily or genera indicate statistical support above 80% SH-aLRT and 90% ultrafast bootstrap. The scale represents amino acid substitutions per site. **B)** Likelihood mapping (treelikeness) of paramyxoviruses V protein sequences

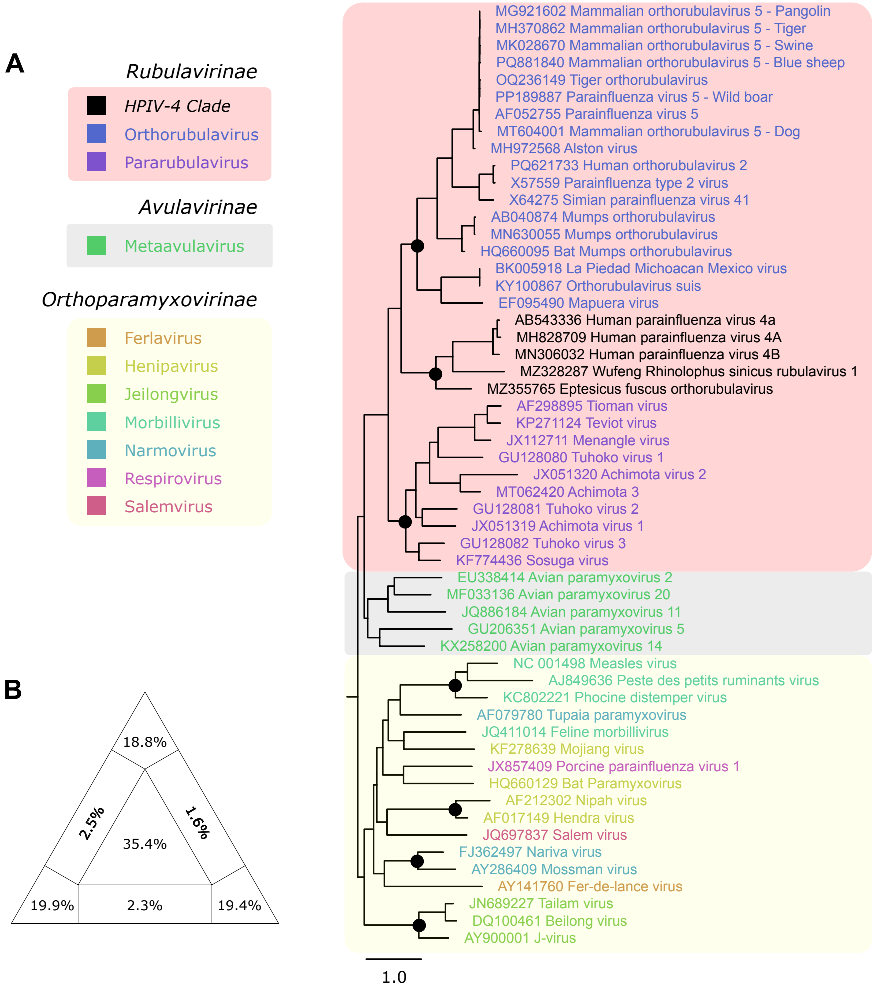

Supplementary Figure 9. Likelihood mapping (*treelikeness*) of paramyxovirus amino acid sequences

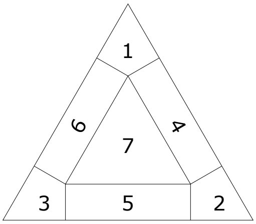

Note: The graphical visualization represents different levels of support for tree topologies and evolutionary models, showing how likely specific tree configurations are given the data and model assumptions. Quartets falling into the three corners (1, 2, and 3) are informative and provide well-resolved phylogenies. Those in three rectangles (4, 5, and 6) are partly informative, and those in the center (7) are uninformative (star-like phylogenies).
Ideally, we want a high number of fully resolved quartets and low number of unresolved quartets. (additional information: Strimmer and Haeseler, Proc. Natl. Acad. Sci. U.S.A. 1997)

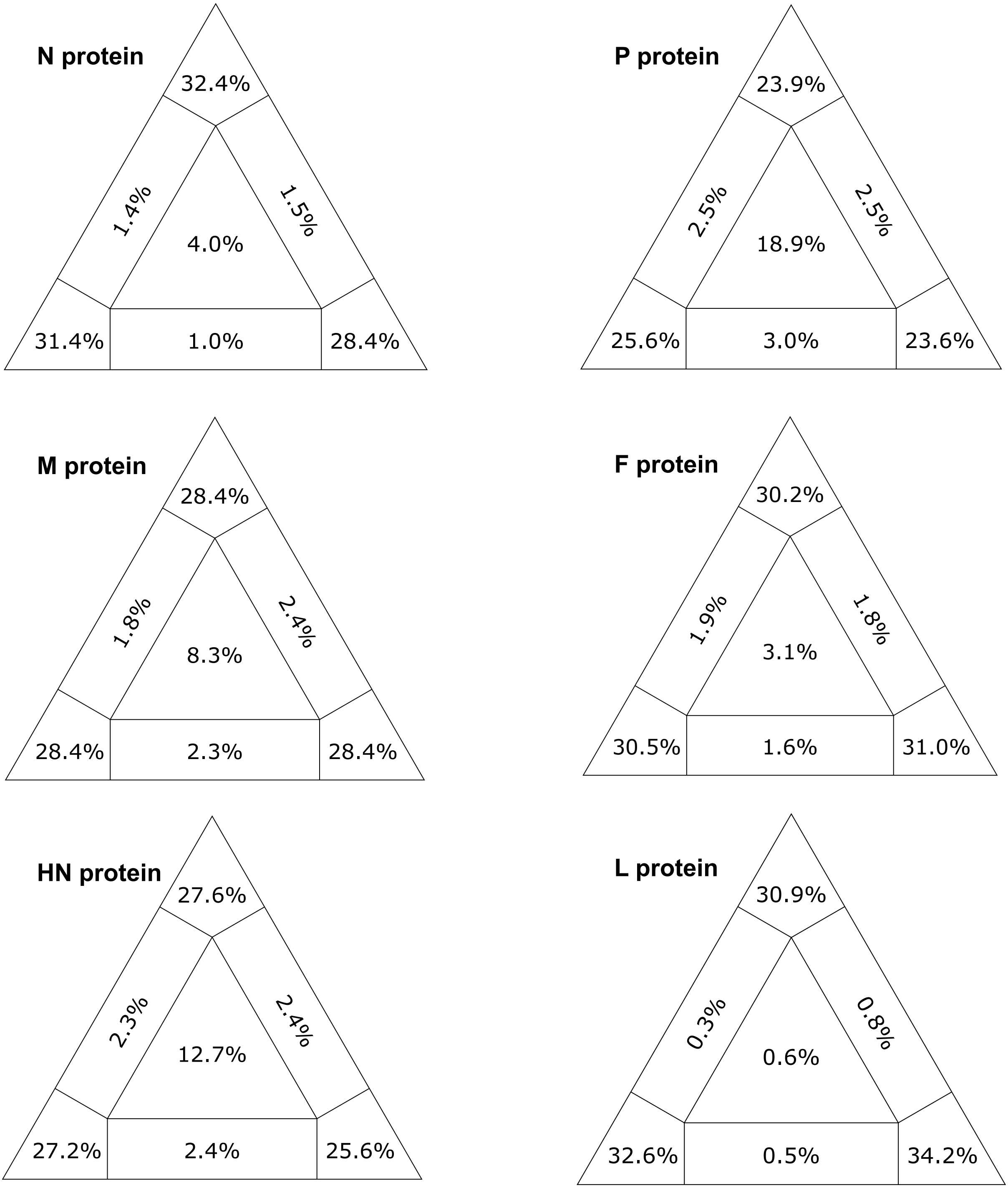

Supplement: msaf217_Supplementary_Data [file msaf217_supplementary_data.zip › SupplementaryFigures.pdf]
